# Hematological Changes in Platelet Apheresis Donors: An Observational Study

**DOI:** 10.7759/cureus.73907

**Published:** 2024-11-18

**Authors:** Anjali Sharma, Satish Arakeri, Rahul Kanungo, Sajal Pagi

**Affiliations:** 1 Pathology, Shri B M Patil Medical College Hospital &amp; Research Centre, BLDE (Deemed to be University), Vijayapura, IND

**Keywords:** donor, hematocrit, platelet, plateletpheresis, single donor platelets

## Abstract

Background

The demand for platelet concentrates is increasing for treating patients in clinics such as oncology. Single donor platelets (SDPs) collected through apheresis offer a lower risk of transfusion-related complications.

Aim

This observational study aims to evaluate hematological changes in donors before and after platelet donation via apheresis, shedding light on donor safety and eligibility criteria.

Methods

This was a pilot study involving 30 plateletpheresis procedures. Hematological parameters were measured before and after donation, including platelet count and hematocrit.

Results

All donors were male, with an age range of 18-45 years. Blood group distribution among donors was O+ve (12), A+ve (6), B+ve (5), AB+ve (4), B-ve (1), O-ve (1), and AB-ve (1). The mean fall in platelet count after donation was 50,833/mm^3^. The overall mean fall in hematocrit was 1.16%, with a range of 0.5-2%. For donors with a platelet yield of 3.0x10^11^, the average hematocrit reduction was 1.13%, whereas for donors with a yield of 3.5x10^11^, the reduction was 1.56%.

Conclusion

Automated cell separators have significantly improved SDP quality and collection efficiency. The correlation between platelet yield and pre-donation platelet count underlines the importance of personalizing collection parameters.

## Introduction

Platelets collected using an apheresis cell separator are commonly referred to as single donor platelets (SDPs). In medical settings, SDP transfusions are typically recommended for preventing and treating bleeding in patients with low platelet counts or issues with platelet function [1].

The demand for platelet concentrates is consistently rising, especially in specialized clinics like oncology, clinical hematology, critical care medicine, hepatology, and transplant units [2]. Patients who have undergone multiple blood transfusions previously and show platelet refractoriness account for 28%-34% [3]. In contrast to random donor platelets (RDPs) obtained from whole blood, SDPs offer a greater number of platelets to the recipient, a lower risk of transfusion-related transmitted infections, and alloimmunization, platelet refractoriness, and other adverse transfusion-related events [4]. 

There is a growing preference for utilizing SDPs to support patients with thrombocytopenia. The collection of SDPs through the apheresis procedure, known as plateletpheresis, is generally regarded as safe for donors and has minimal complications [5]. 

The new generation cell separators allow the easy separation of platelets with minimal manipulation [6]. A variety of apheresis machines are commercially available, operating on the principle of centrifugation. Extensive research has shown that these machines are user-friendly and donor-friendly, ensuring the optimization of platelet quality [7]. 

In many centers, unfortunately, there have been cases of donors experiencing mortality. This underscores the importance of not just focusing on complications, but also closely monitoring changes in laboratory test indicators and the overall well-being of donors post-donation [8].

Examining the safety of apheresis donors is crucial in the field of blood donation. This study focuses on monitoring hematological changes in apheresis donors, providing insights into the physiological impact of these procedures. The findings could have substantial implications for donor care, eligibility criteria, and the overall safety of apheresis donations, potentially raising the standards of care for these generous individuals who contribute vital blood components to patients in need.

Aim of the study

To evaluate changes in hematological parameters like platelet count and hematocrit before and after platelet donation through the apheresis procedure.

## Materials and methods

This pilot study included 30 plateletpheresis SDP procedures conducted from August 2023 to October 2023. Institutional Ethical Committee, BLDE (Deemed to be University), Shri B M Patil Medical College, Hospital & Research Centre issued approval BLDE(DU)/IEC/613/2022-23, dated August 26, 2022. All procedures were performed on eligible donors who provided informed consent.

Donor selection

Following registration, all donors underwent screening for age, weight, blood group, medical history, drug history, vein condition, and other selection criteria. Donors who met the initial screening criteria had whole blood samples collected for mandatory laboratory screening as per national guidelines for plateletpheresis.

Donor sampling

Whole blood samples in ethylenediaminetetraacetic acid (EDTA) and clotted vials were collected before the plateletpheresis procedure. Hematological parameters such as platelet count (PLT), hemoglobin (Hb), hematocrit (Hct), and white blood cell (WBC) count were measured using a calibrated automated cell counter (5-part CBC Analyser). Blood group and antibody screening were confirmed, followed by testing for infectious markers including anti-HIV 1 & 2, anti-HCV (hepatitis C virus), HBsAg (hepatitis B surface antigen), syphilis, and malaria. Anti-HIV 1 & 2, anti-HCV, and HBsAg tests were conducted using the automated VITROS ECiQ immunodiagnostic system (QuidelOrtho Corporation, San Diego, CA, USA) based on enhanced chemiluminescence technology. Syphilis was tested using rapid qualitative immunochromatography, while malaria antigens for *Plasmodium falciparum* and *Plasmodium vivax* were assessed via rapid qualitative chromatographic immunoassay.

Procedure and donor safety

Only donors who passed the screening test were selected for the plateletpheresis procedure. Plateletpheresis procedures were conducted using the Trima accel cell separator. All plateletpheresis procedures were conducted following the manufacturer's instructions and departmental standard operating procedures (SOPs) using recommended apheresis kits. The endpoint of each procedure was determined by the target platelet yield per unit. Donor information, including name, registration number, blood group, age, gender, weight, hematological values, and plateletpheresis procedure details, such as kit information, total blood volume processed, anticoagulant (CPDA-1) volume used, procedure time, blood flow rate, and collection efficiency of machines (PLT yield/total volume of blood processed), was recorded for each procedure in the procedure register. All donors were given prophylactic oral calcium (1000 mg) and were provided with a detailed explanation of the procedure before its commencement. Donors were encouraged to report any discomfort during or after the procedure to the apheresis team. Any donor adverse reactions were managed according to departmental SOPs and documented accordingly.

Quality control of single donor platelets

Approximately 1 ml samples from each bag were collected in EDTA vials after thorough segment stripping to ensure a representative product from the bag. All samples were mixed thoroughly and subjected to quality parameter measurements such as volume, Hct, PLT, RBC, and WBC counts.

## Results

**Figure 1:**
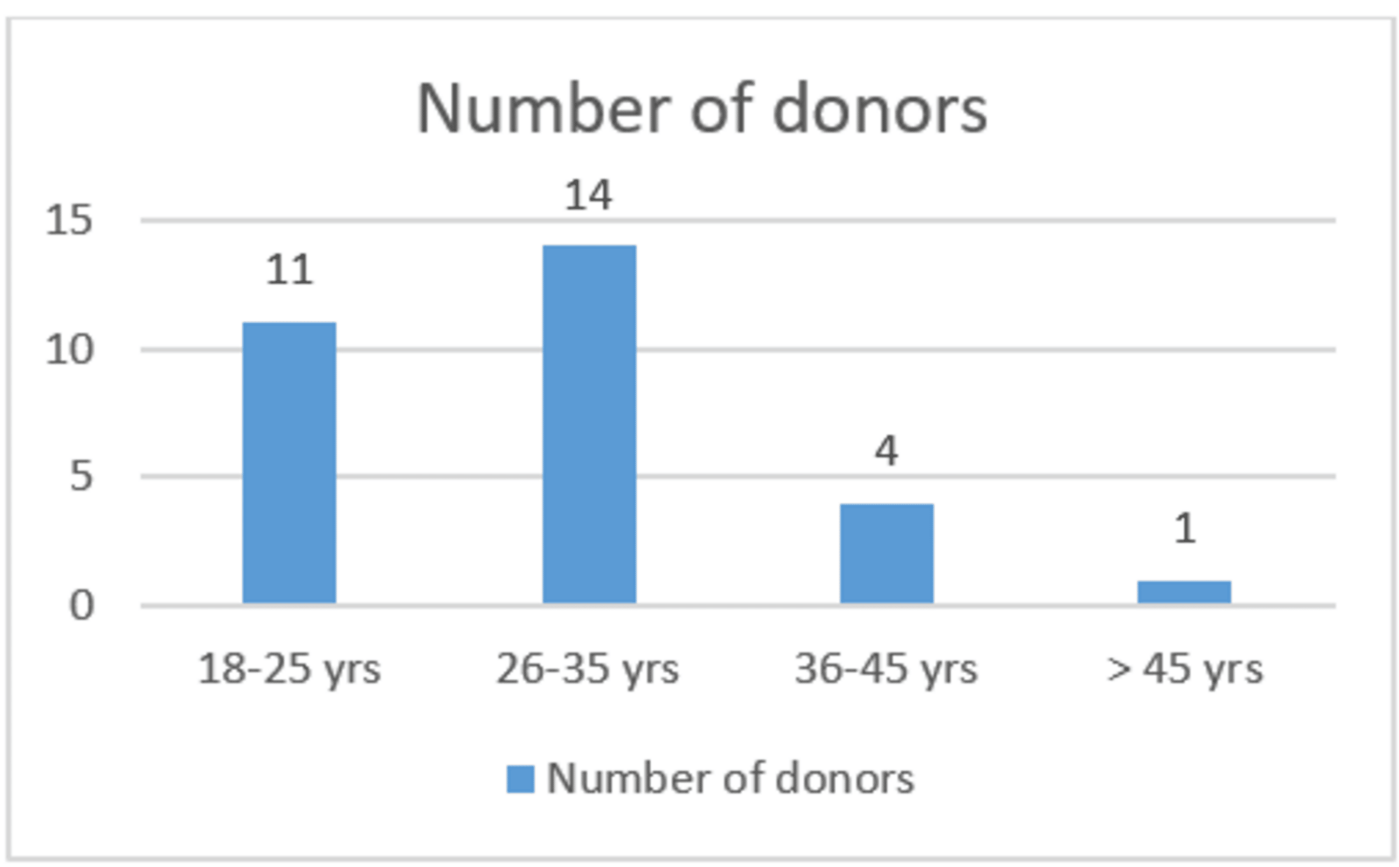
Distribution of donors according to age.

As this study was a pilot study, a sample size of 30 male donors was selected, with age distribution as follows: 11 donors in the 18-25 years range, 14 donors aged 26-35 years, four donors aged 36-45 years, and one donor older than 45 years (Figure 1).

**Figure 2:**
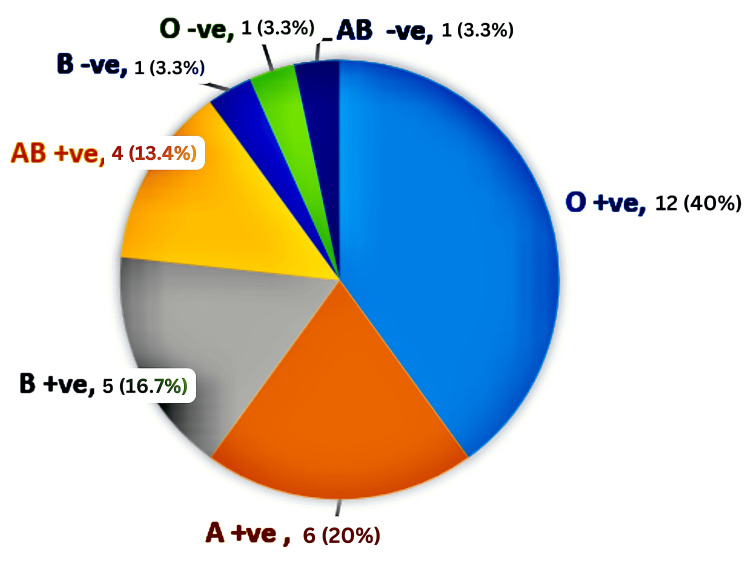
Distribution of donors according to their blood group.

The mean weight of the donors was 73.53 kg, and the average height was 170.7 cm. Blood group distribution revealed 12 donors with O+ve, 6 with A+ve, 5 with B+ve, 4 with AB+ve, 1 with B-ve, 1 with O-ve, and 1 with AB-ve (Figure 2).

Platelet variation after donation

The total platelet count before donation exhibited a range of 223,000-493,000/mm^3^. Following donation, the overall fall of platelet count was 35,000-66,000/mm^3^, with an average fall of 50,833/mm^3^. The reduction in platelet count was directly proportional to the targeted platelet yield. For donors with a platelet yield set at 3.0x10^11^, the platelet fall ranged from 35,000-64,000/mm^3^, with an average reduction of 48,095/mm^3^. Meanwhile, for those with a yield set at 3.5x10^11^, the platelet fall ranged from 51,000-66,000/mm^3^, with an average reduction of 57,220/mm^3^ (Table 1). 

**Table 1:** Variation in platelet count as per platelet yield.

Platelet yield	No. of donors	Range of platelet count before donation	Range of platelet fall after donation	Overall mean fall in platelet count
3.0x10^11^	21	2,23,000 - 4,93,000/mm^3^	35,000 - 64,000/mm^3^	48,095/mm^3^
3.5x10^11^	9	2,25,000 - 4,21,000/mm^3^	51,000 - 66,000/mm^3^	57,220/mm^3^

Hematocrit variation after donation

The range of hematocrit before donation spanned 37.7%-49.5%. Post-donation, the overall fall in hematocrit was 0.5-2%, with a mean reduction of 1.16%. For donors with a platelet yield set at 3.0x10^11^, the hematocrit fall ranged from 0.8-2%, with an average reduction of 1.13%. On the other hand, for those with a yield set at 3.5x10^11^, the hematocrit fall ranged from 0.5-1.6%, with an average reduction of 1.56% (Table 2).

**Table 2:** Variation in hematocrit as per platelet yield.

Platelet yield	No. of donors	Range of hematocrit before donation	Range of hematocrit fall after donation	Overall mean fall in hematocrit
3.0x10^11^	21	37.7% - 48.0%	0.8% - 2%	1.13%
3.5x10^11^	9	38.3% - 49.5%	0.5% - 1.6%	1.56%

The study observed a notable reduction in platelet count following apheresis, averaging 50,833/mm^3^, with variations linked to the targeted platelet yield. Specifically, donors targeted for a yield of 3.0x10^11^ exhibited a mean platelet reduction of 48,095/mm^3^, while those targeted at 3.5x10^11^ showed a higher mean reduction of 57,220/mm^3^. Hematocrit levels displayed a mean decrease of 1.16%, with donors at a 3.0x10^11^ yield experiencing a 1.13% reduction and those at 3.5x10^11^, a slightly greater decrease of 1.56%. These findings underscore the direct relationship between platelet yield and hematological parameter changes.

## Discussion

This pilot study, focusing on the evaluation of changes in hematological parameters pre- and post-platelet donation through the apheresis procedure, observed a significant reduction in platelet count by an average of 50,833/mm^3^. However, it is considerably less than the post-apheresis decrease reported by Enein et al., who observed a decrease of 53.6 ± 26.3/mm^3^, suggesting variability in the impact of apheresis across different settings and methodologies [9].

The targeted platelet yield in our study was set at 3.0 x 10^11^, which is contrasted by the yields reported in other studies. For instance, Hitzler reported a mean platelet yield of 4.5 ± 0.8 x 10^11^/L, and Chopra et al. found the mean platelet yield in single donor platelets (SDP) to be 4.09 ± 1.15 × 10^11^ [10,11]. These discrepancies highlight differences in procedural efficiencies and donor selection criteria across studies [10,11]. 

Our study's findings on the direct proportionality between the reduction in platelet count and the targeted platelet yield mirror the significant correlation between pre-donation platelet count and yield observed by Chopra et al. and the positive correlation reported by Enein et al. between platelet pre-donation count and yield (r = 0.512) [9,11]. This reinforces the predictive value of pre-donation platelet counts for yield outcomes [9,11]. 

In terms of hematocrit changes, our study noted a mean reduction of 1.16% post-donation. This is in line with the findings of Rajput et al., who reported a non-significant decline in hematocrit (p = 0.44), indicating that the impact of plateletpheresis on hematocrit levels is generally minimal and transient [12]. This suggests that the procedure is safe in terms of maintaining red blood cell volume [12]. 

Thokala et al.'s observation of a progressive post-procedure increase in platelet count across all groups underscores the body's ability to recover post-donation [13]. Our study did not measure platelet count recovery over time, presenting an area for future research to better understand the timeline and mechanisms of hematological recovery post-apheresis [13]. 

This reduction in platelet count is within the range but notably distinct from the findings of Syal et. al., who documented a decrease in post-donation mean platelet count of 70,800/mm^3^ [14]. Moreover, the increase in post-procedural mean hemoglobin levels reported by Syal et al., with a statistically significant increase of 0.14 g/dl, contrasts with the expectation of stable or declining levels post-donation. This observation may indicate procedural or physiological differences that merit further investigation [14]. 

Comparatively, our study enriches the existing literature by providing detailed insights into the hematological impact of plateletpheresis in a specific donor population. It also highlights the need for further research to explore the influence of factors such as blood group on yield outcomes, as our study did not find a significant impact, aligning with Ugwu et al.'s findings [15].

A key difference underscored by our findings is the direct correlation between targeted platelet yield and the magnitude of platelet reduction, a detail that complements the existing literature by providing a focused examination of the physiological implications of yield targets on donor safety and recovery. Furthermore, our study adds to the discourse on the safety of plateletpheresis by documenting minimal changes in hematocrit levels, reinforcing the procedure's safety profile as noted by Rajput et al. but within the context of a different target yield and donor demographic [12].

The key takeaway from our study is the affirmation of plateletpheresis as a safe procedure for donors, with predictable hematological outcomes that are directly influenced by the target platelet yield. This insight is crucial for optimizing donor selection criteria and procedural settings to balance the demands of platelet supply with donor safety and well-being. Our findings suggest that while higher yield targets are achievable and have been documented in the literature, a conservative approach to yield targeting can still meet clinical needs without compromising donor health, offering a strategic perspective for blood donation centers aiming to maximize both donor safety and the efficiency of platelet collection.

Limitations of the study

This study's limitations include its small sample size of 30 male donors, which restricts the generalizability of findings across a more diverse donor population. Additionally, only immediate post-donation hematological changes were examined, leaving the long-term impacts on donor health unassessed. Future studies with larger, more diverse cohorts and extended follow-up would provide a more comprehensive understanding of the hematological and safety implications of plateletpheresis.

## Conclusions

Automated cell separators have significantly improved the efficiency and quality of single donor platelet collections, enabling a controlled reduction in platelet count and hematocrit that aligns with safe donor thresholds. This study reaffirms plateletpheresis as a generally safe procedure for donors, with predictable hematological outcomes that can be optimized through appropriate yield targets. The findings support the continued use of apheresis in clinical settings, emphasizing the importance of balancing platelet yield with donor safety to encourage ongoing participation in donation programs.
